# Supplementary material for: Temperature heterogeneity correlates with intraspecific variation in physiological flexibility in a small endotherm
Source: Nat Commun. 2021 Jul 20;12:4401. doi: 10.1038/s41467-021-24588-6 (PMC8292308; doi:10.1038/s41467-021-24588-6)
Supplement: Supplementary file 1 — Supplementary Information [file 41467_2021_24588_MOESM1_ESM.pdf]

Supplemental Materials

Table S1. AIC values from models depicting the effects of 8 environmental variables on *Junco* M_{sum} assayed in the field: minimum temperature (T_{min}), maximum temperature (T_{max}), precipitation (prcp), daylength (dayl), water vapor pressure (vp), shortwave radiation (srad), daily temperature range (T_{d_range}), and elevation. Weather variables are the mean value at the site of capture for the indicated number of days preceding capture (except for elevation, which did not change over time). All models include M_b and taxon as covariates, and continuous variables were standardized (k = 3, n = 335 individuals). Null model ($M_{sum} \sim M_b$ + taxa): k = 2, AIC = 1022.92. Best model is shown in bold.

Days	T_{min}	T_{max}	prcp	dayl	vp	srad	T_{d_range}	elev
7	1006.34	985.72	994.82	1020.39	1001.31	979.67	965.49	1023.35
8	1007.66	986.74	991.20	1020.66	1001.08	976.90	**960.94**	
9	1009.70	989.48	987.67	1020.92	1001.20	983.15	966.85	
10	1012.98	992.92	987.74	1021.18	1001.86	989.67	970.99	
11	1015.44	996.37	990.25	1021.43	1002.45	994.30	970.69	
12	1016.52	998.79	989.73	1021.68	1001.71	997.31	969.50	
13	1017.90	1000.26	988.61	1021.92	1000.05	999.63	970.67	
14	1019.17	1001.10	989.27	1022.16	1000.62	1001.15	968.15	

Table S2. Primer sequences used for generating RAD-Seq libraries following ref.[1].

MseI1: 5' GCAGAAGACGGCATACGAGCTCTTCCGATCTG 3'
MseI2: 5' TACAGATCGGAAGAGCTCGTATGCCGTCTTCTGCTTG 3'

Illpcr1 (Forward): 5' A*A*TGATACGGCGACCACCGAGATCTACACTCTTTCCCTACACGACGCTCTTCCGATCT 3'
Selective Illpcr2 (Reverse): 5' C*A*AGCAGAAGACGGCATACGAGCTCTTCCGATCTGTAAG 3'

Figure S1. Relationship between flexibility in M_{sum} in the field (measured as the slope of the reaction norms shown in Figure 1) and the thermal heterogeneity of the environment (approximated as the average annual temperature range from the WorldClim dataset). Each dot represents a taxon; colors correspond to Figure 1 (*J. h. caniceps* = blue; *J. h. hyemalis* = red; *J. h. mearnsi* = green; *J. h. oreganus* group = orange; *J. p. palliatus* = yellow). The dashed line represents a regression fit to four populations (excluding *J. h. mearnsi*): $R^2 = 0.92$, $\beta = 0.02 \pm 0.004$, $p = 3.9 \times 10^{-2}$, $n = 4$ populations.

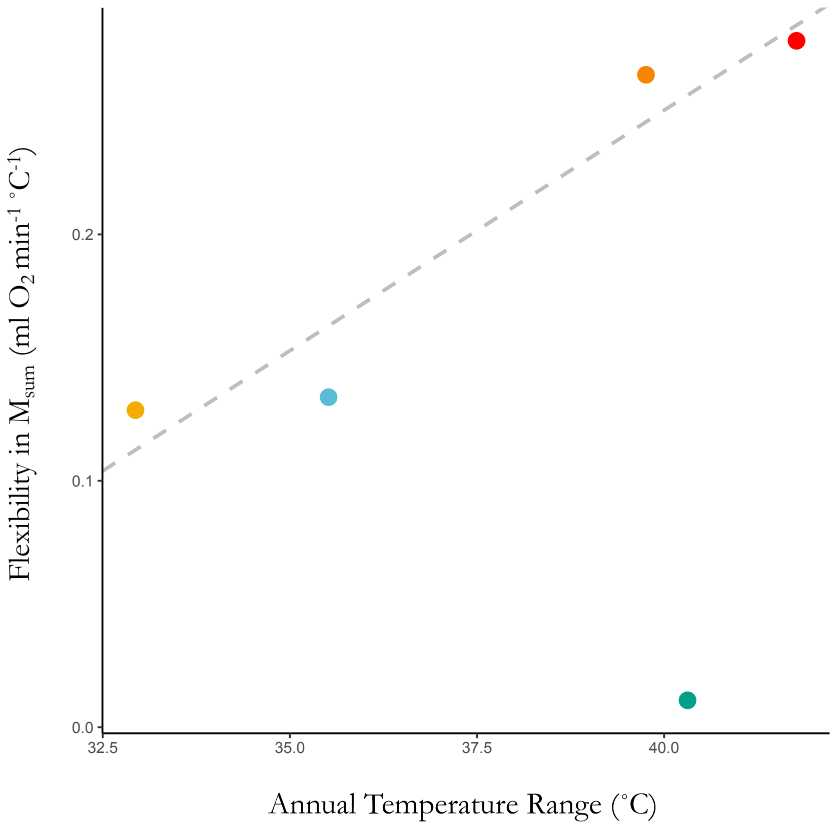

Figure S2. Variance in M_{sum} among 82 wild-caught *J. h. montanus* from Montana decreased from σ = 2.24 to σ = 0.52 after a six-week adjustment period under common conditions in the lab (i.e. 18°C with 10 h:14 h light dark, *ad libitum* food and water). Boxplots show the median values (midline), the 25th and 75th percentiles (lower and upper margins of the box), the minimum and maximum values ≤ 1.5 × IQR from the box margin (whiskers), and outlier values (open circles). M_{sum} values collected within 24h of capture are included in Source Data file within the *J. h. oreganus* group. M_{sum} values after 6 wk adjustment period are published in ref.[2].

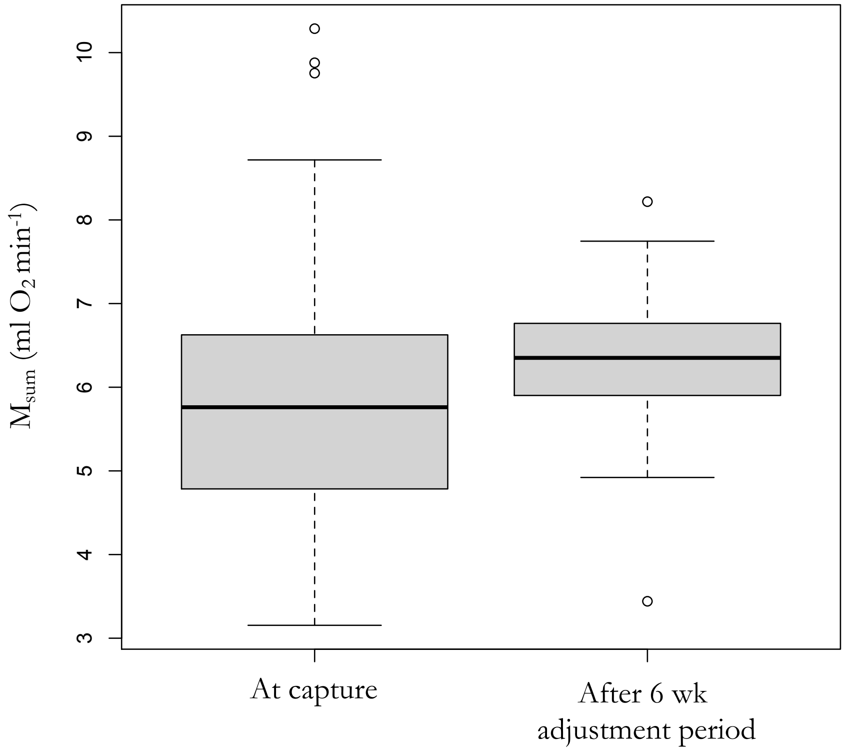

REFERENCES

1. Parchman, T. L. *et al.* Genome-wide association genetics of an adaptive trait in lodgepole pine: association mapping of serotiny. *Molecular Ecology* **21**, 2991–3005 (2012).
2. Stager, M., Senner, N. R., Tobalske, B. W. & Cheviron, Z. A. Body temperature maintenance acclimates in a winter-tenacious songbird. *J Exp Biol* **223**, jeb221853 (2020).
